# Bradycardia-Induced Torsades de Pointes in Atrioventricular Block

**DOI:** 10.7759/cureus.37507

**Published:** 2023-04-12

**Authors:** Pradnya Brijmohan Bhattad, Anil Jha, Richard Wholey

**Affiliations:** 1 Cardiovascular Medicine, Saint Vincent Hospital, University of Massachusetts (UMass) Chan Medical School, Worcester, USA

**Keywords:** polymorphic ventricular tachycardia, sinus bradycardia, isoproterenol, high-grade atrioventricular block, prolonged qt interval, torsades de pointes

## Abstract

Bradycardia is known to prolong QT interval. Persistent bradycardia and high-grade atrioventricular (AV) block may lead to persistently prolonged QTc interval with a risk for life-threatening ventricular arrhythmias, which needs addressing the underlying cause. We present the case of a patient with persistent sinus bradycardia with a high-grade AV block leading to persistently prolonged QTc without any reversible etiology that resulted in torsades de pointes. The underlying treatment involved shortening the QTc by increasing the heart rate to prevent any further episodes of polymorphic ventricular tachycardia.

## Introduction

Bradycardia is a well-known risk factor predisposing to torsade de pointes (TdP) primarily due to prolonged QT interval [1-2]. The heart rate in the repolarization time has an inverse relationship, probably accounting for bradycardia-induced QT prolongation. TdP-induced bradycardia has been described as associated with several conditions, such as drug-induced, atrioventricular (AV) block, vasovagal episodes, and hypothyroidism [3-6]. Our case presents a patient with persistent sinus bradycardia with a high-grade AV block leading to persistently prolonged QTc without any reversible etiology that resulted in TdP.

## Case presentation

**Figure 1:**
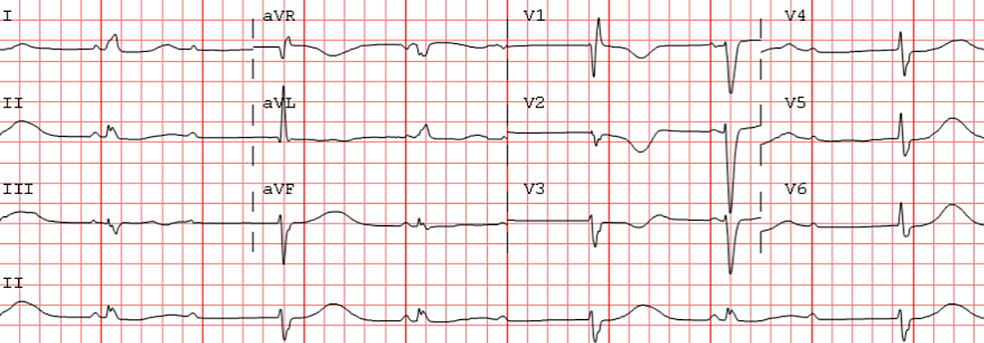
ECG showing sinus bradycardia with an AV 2:1 Mobitz type II block with ventricular escape rhythm.

An 86-year-old female with no significant past medical history presented to the ER complaining of lightheadedness preceding a syncopal event. She reported experiencing lightheadedness, following which she lost consciousness and had an unwitnessed fall, after which she was brought to the ER for further evaluation. She did not recall any of her symptoms immediately before her syncopal event outside of feeling lightheaded. She regained her consciousness at some point when she was being transported to the ER. At the time of her presentation in the ER, she was hemodynamically stable; her heart rate was in the 30s-40s/minute, her blood pressure was 144/68 mmHg, and her oxygen saturation of 98% on room air. A complete blood cell count, chemistry panel, electrolytes, magnesium, potassium levels, and thyroid function studies were noted in the normal reference range at the presentation time. She took no medications at home. She did not take any herbal agents, over-the-counter medications, or supplements. She did not use any street drugs or illicit substances. An ECG at the time of presentation showed a second-degree AV block (Figure 1). 

**Figure 2:**
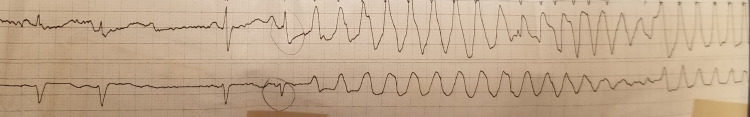
Telemetry strip 1 shows long QTc with a long R-R interval followed by a short R-R interval in a pattern of alternating long-short R-R intervals. There is an R on T PVC preceding the onset of polymorphic ventricular tachycardia. The episode of polymorphic ventricular tachycardia here is nonsustained and terminates spontaneously. The patient was completely asymptomatic during this episode. She received a bolus of IV magnesium immediately after this was noticed on telemetry. PVC: Premature vntricular complex.

**Figure 3:**
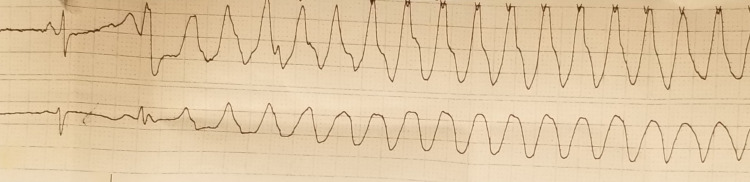
Telemetry strip 2 shows similar findings as strip 1. This was a sustained episode of torsades de pointes which required the institution of ACLS protocol. ACLS: Advanced cardiac life support.

**Figure 4:**
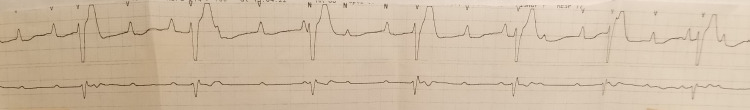
Telemetry strip 3 shows a complete heart block with AV dissociation noted after resuscitation. AV: Atrioventricular.

She was hospitalized for further monitoring. She continued having persistent bradycardia with a heart rate of 30s/min and a high-grade AV block throughout continuous telemetry monitoring. She was scheduled for a permanent pacemaker implantation as no reversible etiology of her high-grade AV block was evident. She was noted to have prolonged QTc interval in the setting of continued bradycardia. While she was awaiting permanent pacemaker implantation, she developed an episode of bradycardia-induced polymorphic ventricular tachycardia for which advanced cardiac life support (ACLS) protocol was instituted (Figures 2-4).

She received chest compressions and underwent defibrillation per ACLS protocol, after which return of spontaneous circulation was attained, and she was back to her baseline sinus rhythm with high-grade AV block with a baseline heart rate in 30s/minute. She was administered IV magnesium bolus and was started on isoproterenol fusion with a goal target heart rate in the 80s-90s/minute due to the sustained episode of bradycardia-induced TdP and the patient being in continued high-grade AV block with significantly prolonged QTc interval while she was awaiting permanent pacemaker implantation. After three hours of her episode of sustained polymorphic ventricular tachycardia, the patient underwent permanent pacemaker implantation, and her isoproterenol infusion was discontinued. She remained hemodynamically stable without any further ventricular arrhythmia episodes after permanent pacemaker implantation. She was discharged home and will be followed as an outpatient.

## Discussion

TdP is a life-threatening condition characterized by polymorphic ventricular tachycardia associated with prolonged QT interval at baseline. Significantly increased transmural depression of repolarization (TDR) acts as a reentrant substrate to initiate and continue TdP episodes at the contiguous myocardial sites. Descending limb of the T wave represents TDR on the EKG, which is considered vulnerable to initiating Torsades [7]. It has been seen that patients with heart block present with significantly longer QT intervals compared to those with sinus bradycardia at similar heart rates [8]. As the action potential duration prolongs, this can occur due to both bradycardia-dependent depressions of electrogenic sodium pumping and more complete inactivation of IK, which may cause early after depolarizations [9]. Low ventricular rates are also associated with submaximal activation of ITO, causing shifts of the plateau of the action potential to voltage levels at which Ca2+ window current availability increases.

Contrary to this, higher heart rates oppose this phenomenon and prevent early after depolarization and TdP [10]. We excluded all reversible causes for AV block in our case. There is no robust evidence-based guideline for implantation of pacemaker insertion in these patients. However, a few clinical scenarios, such as symptomatic chronic AV block with a narrow QRS escape rhythm noted the first time and persistent third-degree AV block with an escape rate greater than 40 bpm without cardiomegaly, favor pacemaker implantation as a reasonable approach [11].

## Conclusions

In conclusion, this case report highlights that patients with AV blocks and bradycardia should always be monitored closely to recognize life-threatening arrhythmia in the setting of TdP. An extensive workup to exclude any reversible cause and decide on appropriate therapy is paramount.
